# Microtubule cytoskeleton-disrupting activity of MWCNTs: applications in cancer treatment

**DOI:** 10.1186/s12951-020-00742-y

**Published:** 2020-12-14

**Authors:** Lorena García Hevia, Mónica L. Fanarraga

**Affiliations:** Nanomedicine Group, Valdecilla Research Institute-IDIVAL, University of Cantabria, Herrera Oria s/n, 39011 Santander, Spain

## Abstract

Microtubules and carbon nanotubes (CNTs), and more particularly multi-walled CNTs (MWCNTs), share many mechanical and morphological similarities that prompt their association into biosynthetic tubulin filaments both, in vitro and in vivo. Unlike CNTs, microtubules are highly dynamic protein polymers that, upon interaction with these nanomaterials, display enhanced stability that has critical consequences at the cellular level. Among others, CNTs prompt ectopic (acentrosomal) microtubule nucleation and the disassembly of the centrosome, causing a dramatic cytoskeletal reorganization. These changes in the microtubule pattern trigger the generation of ineffective biomechanical forces that result in migration defects, and ultimately in spindle-assembly checkpoint (SAC) blockage and apoptosis. In this review, we describe the molecular mechanism involved in the intrinsic interference of CNTs with the microtubule dynamics and illustrate the consequences of this effect on cell biomechanics. We also discuss the potential application of these synthetic microtubule-stabilizing agents as synergetic agents to boost the effect of classical chemotherapy that includes spindle poisons (i.e. paclitaxel) or DNA interfering agents (5-fluorouracil)-, and list some of the advantages of the use of MWCNTs as adjuvant agents in preventing cell resistance to chemotherapy.
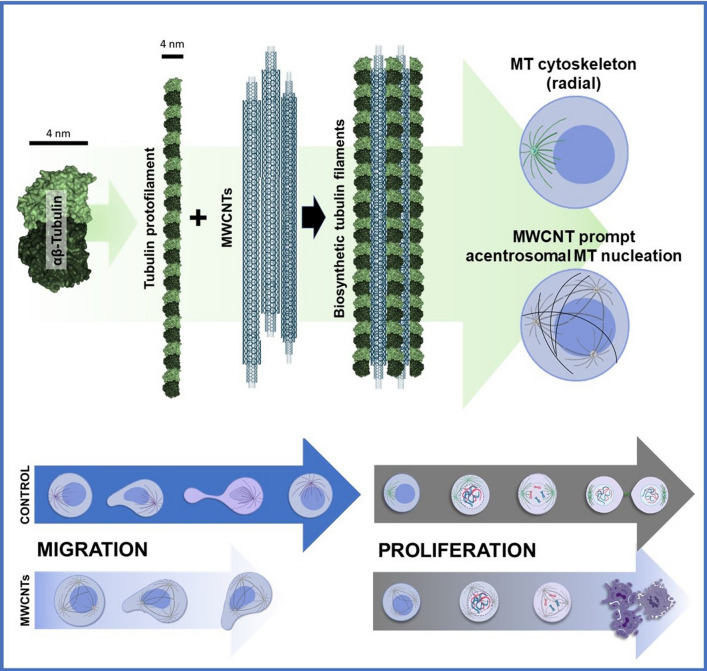

## Carbon nanotubes

Carbon nanotubes (CNTs) are materials with exceptional properties from the physicochemical point of view that are becoming increasingly important in the field of nanobiotechnology. Their reactive surface allows these nanofilaments to capture large amounts of biomolecules on their corona [1–3]. This CNT proteinaceous coating behaves as biological camouflage that endows the nanotubes with the ability to selectively interact with receptor proteins or participating in specific protein-protein interactions [2, 4, 5]. CNTs are also well-known to adsorb many different types of inorganic molecules or drugs [6–16] or nucleic acids [9, 17] all of the great interest in nanomedicine. Finally, at the elementary level, CNTs are pure carbon and this composition makes them highly biocompatible since carbon represents ca. 18% of the composition of the human body.

Another interesting feature of CNTs is their one-dimensional morphology. This property—very attractive from a biotechnological point of view—endows nanotubes with the unique ability to penetrate inside cells and cross tissues. But, from a biotechnological level, this feature represents a double edge sword since, CNTs can penetrate through most biological barriers causing putative potential long-term effects. This fact, in addition to their bio-persistence, are some of the great obstacles that make the use of CNTs in nanomedicine a matter of much debate.

Most studies that investigate the biological consequences of CNTs demonstrate very significant phenotypic effects. These nanofilaments have been reported to trigger the production of reactive oxygen species [18–22], DNA breakage [23–26], chromosomal mal-segregation [27–30], anti-proliferative [31–33] and anti-migratory [18, 34–36] effects, etc. Unfortunately, some early toxicity studies were carried out using aggregated or poorly purified CNTs -containing traces of contaminating metals-, or were produced using unrealistic amounts of the nanotubes. This all resulted in poorly reproducible and unpredictable deleterious biological behaviours, leaving a blurry picture of CNTs toxicity that has not been resolved until recently.

We now know that individualized CNTs (non aggregated) can interfere with many cellular processes, and to what extent the nature, surface properties, and size of the nanotubes are important in this process. One of the greatest cellular effects caused CNTs results from their interaction with intracellular filaments, principally with the DNA, actin, and, above all, with the microtubule cytoskeleton. CNT interactions with these biological polymers have been reported to trigger clastogenic effects (DNA breakage), mitotic aberrations, chromosome missegregation, and migratory defects, all leading to a general cellular malfunction that eventually leads to apoptosis.

**Fig. 1:**
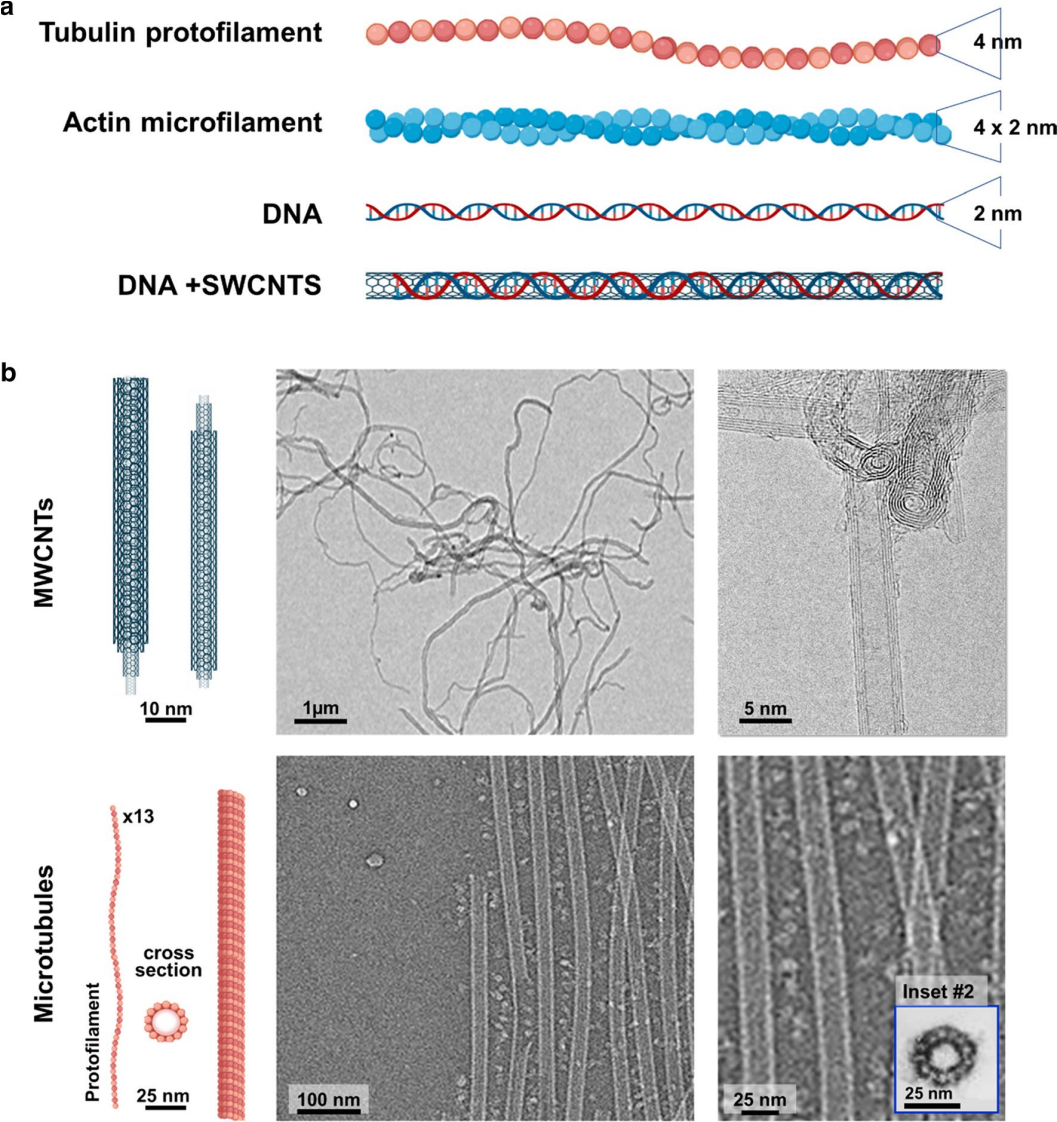
Similarities between intracellular filaments and CNTs. **a** Tubulin protofilaments, actin microfilaments, and DNA have all been reported to interact with CNT. SWCNTs are very similar in size to DNA. This could explain their intrinsic clastogenic (DNA-breaking) effect. **b **MWCNTs display diameters that vary between 4 and 25 nm. MWCNTs and microtubules share many morphological features. Ultrastructurally, MWCNTs and microtubules are very similar. Microtubule SEM image is adapted from Burgess et al. Nat. Comm. (2015) 6:8179

Interestingly, the cellular phenotype produced by different CNTs is not identical. For instance, single-walled CNTs (SWCNTs) have been mostly reported to cause DNA damage [18, 23, 27, 37], while MWCNTs, appear to preferentially interfere with actin [38–40] and tubulin, hindering cellular biomechanics (Fig. 1) [31, 36, 41]. These cellular phenotypes suggest the thickness of the nanotubes could be a significant issue in their interaction with intracellular filaments and, more particularly, with the interaction of the nanotubes with the dynamic polymeric cytoskeletal filaments, such as microtubules or actin microfilaments.

## Microtubules

Microtubules are intracellular tubulin polymers that constitute a major component of cytoskeletal networks in all eukaryotic cells. Microtubules are involved in critical vital functions including DNA segregation during cell division, intracellular vesicular trafficking, and organelle distribution, providing a general structural cellular support, and are pivotal elements in cell migration [42]. The microtubular cytoskeleton is characteristically organized as an intracellular radial network that nucleates in a structure known as the centrosome, close to the nucleus. During cell division, the microtubular network typically reorganizes, first depolymerizing completely, to immediately repolymerize assembling a spindle-shaped structure, the so-called ‘mitotic spindle', responsible for sister cell separation.

Microtubules get their name from their tubular structure [43, 44]. These are 25 nm diameter protein twisted cylinders constituted of 13 protofilaments assembled upon a head-to-tail alignment of αβ-tubulin heterodimers (Fig. 1b). This fact confers microtubules with an intrinsic polarity that results in different properties at both ends of the microtubule [45, 46]. The end containing α-tubulin (Fig. 1b) is located in the centre of the cell in a structure known as the “centrosome” where microtubules nucleate. And the other end, displaying a β-tubulin molecule, is localized in the cell periphery. This microtubule extreme (the so-called `+´ end) is highly dynamic, and it is constantly undergoing cycles of polymerization-depolymerization [46]. This remarkable behaviour, known as `dynamic instability´, increases up to 20 times during mitosis when the microtubule cytoskeleton has to assemble the mitotic spindle [47].

In vivo, microtubule polymerization-depolymerization cycles are tightly regulated by numerous cell factors, mostly microtubule-associated proteins [48] while in vitro polymerization is mostly tubulin-concentration dependent [49]. As an average, tubulin has an intracellular concentration of ca. 5 µM, independently of the stage of the cell cycle. This is cell-dependent but, in general, represents a median of 4.5–5% of the total soluble cellular protein [50, 51].

The implications of the microtubule cytoskeleton in cell division make it a classical target of chemotherapy. Antitumoral drugs interfering with microtubule dynamics, also known as spindle poisons, effectively block cell proliferation, activating the metaphase spindle-check point (SAC), finally unchaining a cell “suicide” effect leading to apoptotic cell death (see below) [47, 52, 53]. The effect of some of these drugs is reviewed in the next sections has the ultimate goal to interfere with the formation of the mitotic spindle, blocking the separation of daughter (or sister) cells, finally triggering apoptosis [52]. These molecules, broadly used in the treatment of cancer, include a series of different compounds that typically target β-tubulin, interfering with the polymerization or depolymerization dynamics of the protofilament thus, behaving either as microtubule-destabilizing or microtubule-stabilizing agents [41].

## MWCNTs are similar to microtubules

**Fig. 2:**
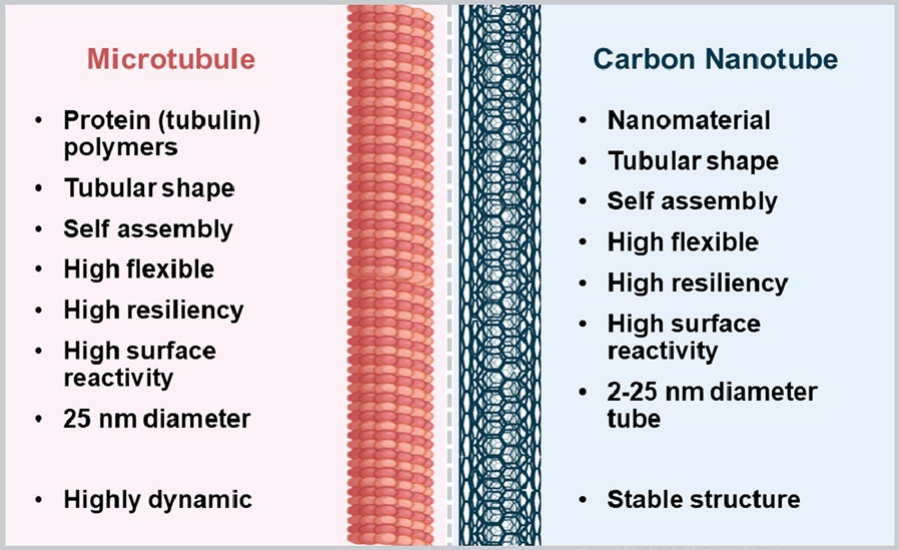
Similarities and differences between microtubules and CNTs

MWCNTs and microtubules share several aspects of their architecture and properties (Fig. 2). Indeed, MWCNTs have been proposed as the technological counterpart of nature’s microtubules [54]. They both have similar dimensions, a tubular morphology constituted of subunits that self-assemble that ensures structural efficiency. Both have analogous physical properties (for example, shear stress, bending stiffness, and Young´s modulus), a highly reactive surface, and both are exceptionally resilient [55, 56]. The greatest difference between these two filaments, which has critical implications in vivo, is their dynamic behaviour. While MWCNTs are stable filaments, microtubules, as reviewed in the previous section, are highly dynamic protein polymers [46].

**Fig. 3:**
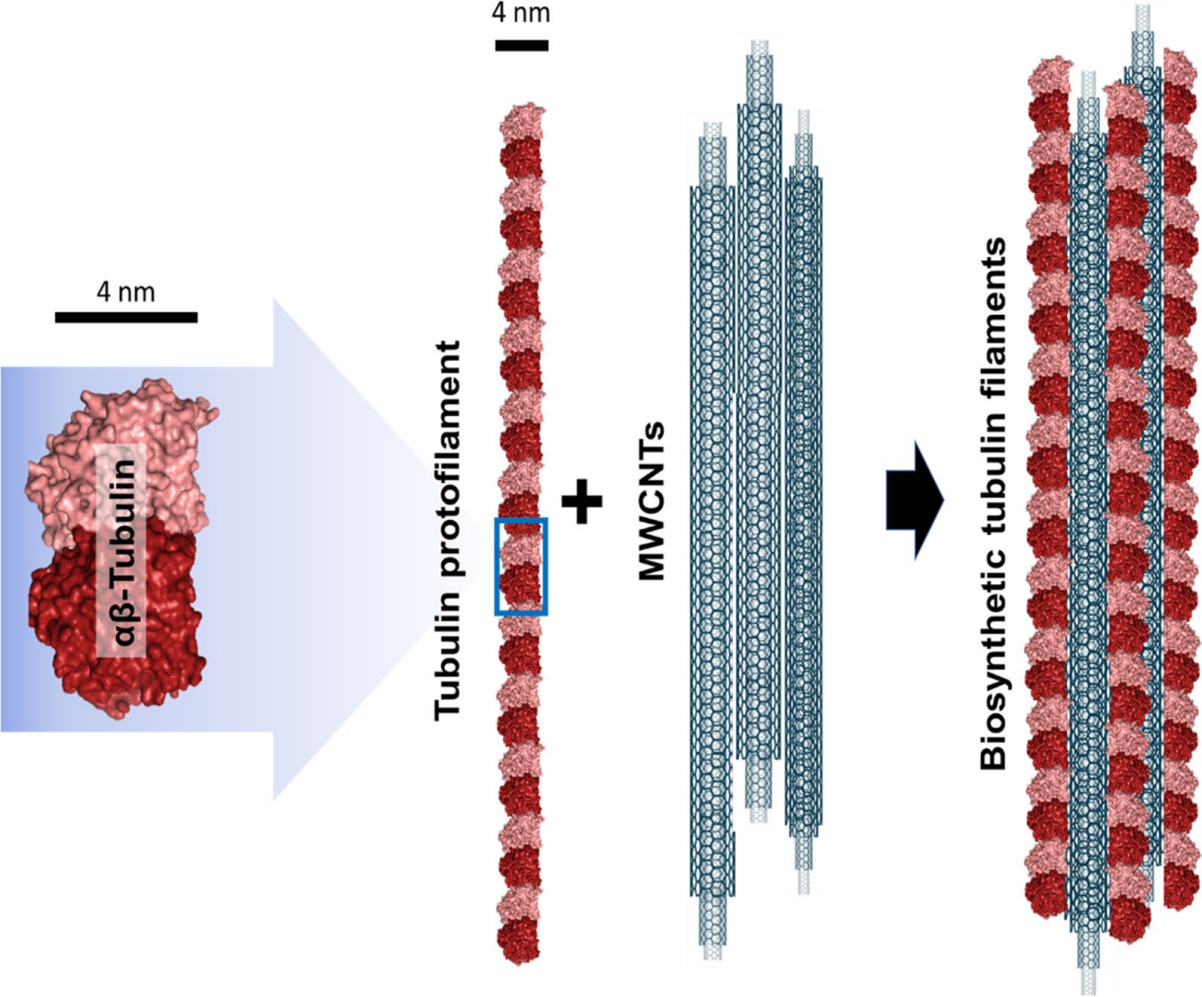
Tubulin association with MWCNTs into biosynthetic filaments. Tubulin heterodimer structure. In the dimer, α-tubulin is colored in dark red and β-tubulin in pink. Head-to-tail tubulin heterodimer alignment assembles protofilaments of 4 nm diameter that intermingle with the MWCNTs and from biosynthetic tubulin filaments

**Fig. 4:**
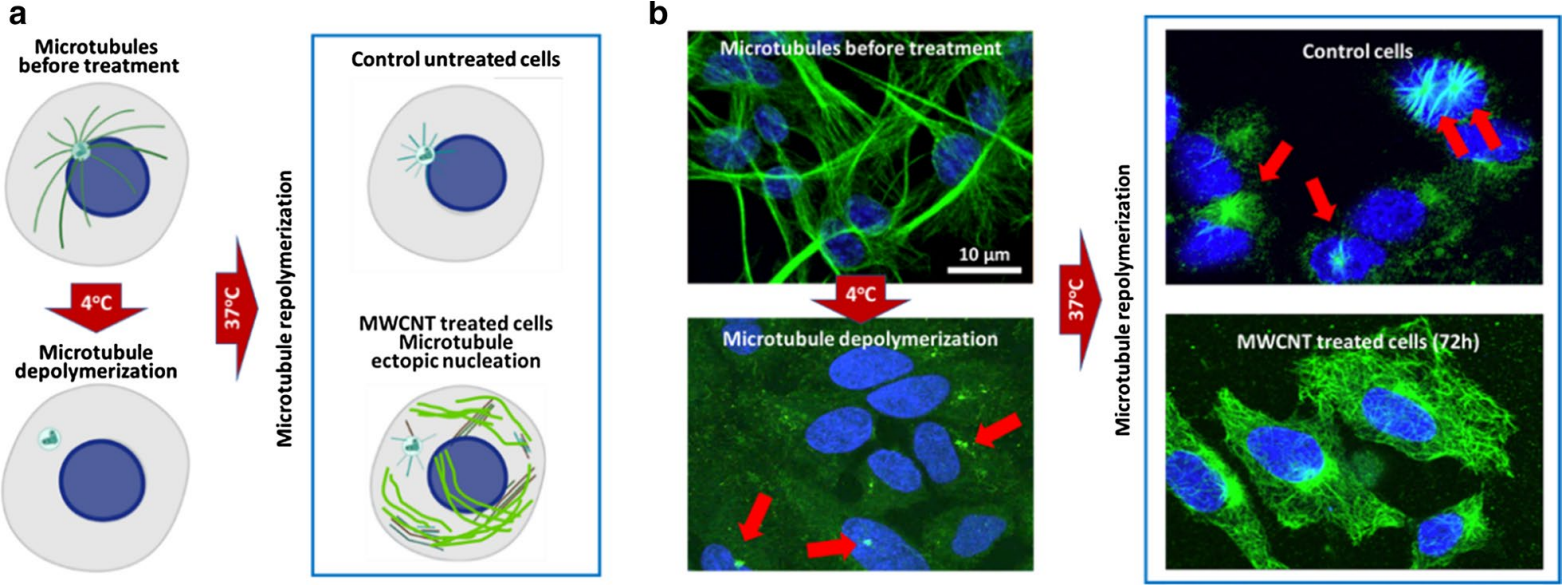
Intracellular MWCNTs prompt ectopic microtubule polymerization. **a** Diagram of the microtubule cold-depolymerization experiment. **b **The microtubule cytoskeleton (labeled in green) is cold-sensible. Upon cell exposure to 4 °C, the microtubules depolymerize and tubulin is only visible at the centrosome (red arrows). Upon return into permissive conditions (37 °C), microtubules regrow from the centrosomes (top, right, arrows). If cells were previous exposed to MWCNTs, tubulin re-polymerizes ectopically all over the cytoplasm. The figure is partially adapted from ref. [31]

Similarities between CNTs and microtubules prompt their association both, in vitro [54, 57] and in vivo [31, 41] into mixed hybrid tubulin polymers (Fig. 3). In vitro upon incubation, tubulin polymerizes on CNTs assembling mixed polymers that allow microtubule motor (dynein–kinesin) “walking” on them, generating functional microtubule-like structures [58, 59]. Intracellularly, CNTs, and more particularly MWCNTs, associate with the cytoplasmic tubulin. Similar to in vitro, tubulin in vivo uses the nanotube surface as a scaffold to polymerize assembling hybrid tubulin nanofilaments [31]. This association, demonstrated by different techniques, is patently observable during microtubule assembly in cold depolymerization-re-polymerization experiments. Upon temperature permissive conditions are applied, tubulin protofilaments polymerized ectopically throughout the cytoplasm, while in untreated cells, microtubules typically nucleate at the centrosome (Fig. 4). The hypothesis is that tubulin polymerization on the nanotube changes the protofilament curvature, stabilizing the now aligned tubulin heterodimers. As a consequence, these hybrid microtubules lose their ability to depolymerize abruptly. The resulting intracellular hybrid disorganized microtubules are quasi-functional but display enhanced stability [31, 32, 41, 57].

**Fig. 5:**
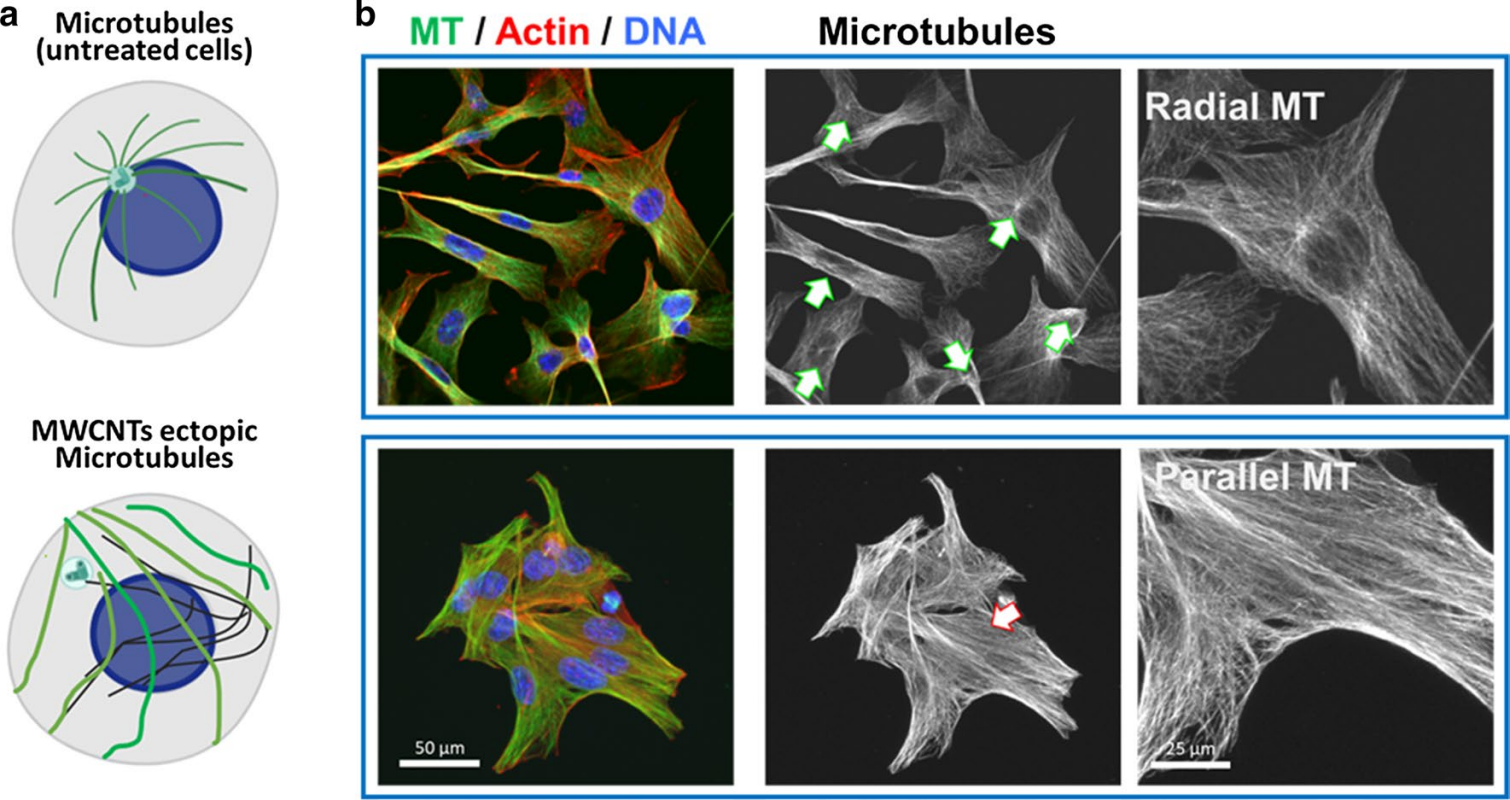
Changes in the microtubule organization in cells treated with MWCNTs. **a **Diagram of the microtubule disposition in control (untreated) cells, and cells treated with MWCNTs. Centrosomes are indicated with green-white arrows.** b** The disassembly of the centrosomes (red-white arrow) and the appearance of parallel microtubules is a consequence of MWCNT-induced microtubule stability. The figure is partially adapted from ref. [35]

**Fig. 6:**
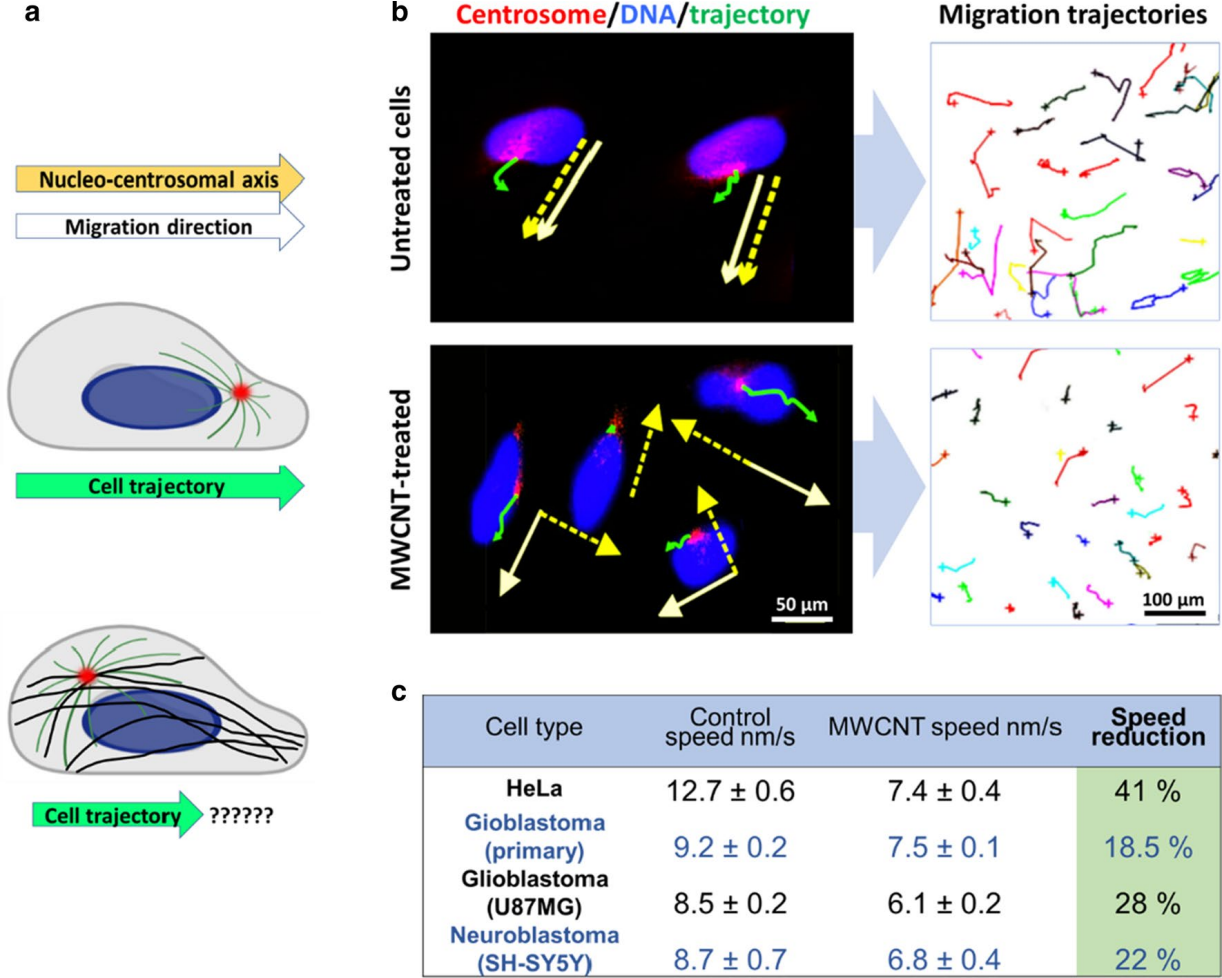
MWCNTs produce an anti-migratory effect on cancer. **a** Migration trajectories of EB1 centrosome-labelled HeLa cells. Cell nuclei are labeled in blue, centrosomes in red and the centrosomal trajectories in green. The migration and the nuclear-centrosome axis directions are indicated in white and yellow, respectively. Cells treated with MWCNTs for 72 h display aberrant migration directions. As a result, their migration trajectories are shorter. **b** Calculated speeds for Hela cells and cancer cells exposed to MWCNTs. A maximum speed reduction was observed for HeLa cells. The figure is partially adapted from ref. [35]

This makes the microtubule nucleation independent of the centrosome, increasing microtubule ectopic nucleation throughout the cytoplasm (tubulin polymerization is no longer radial) resulting in an acentrosomal disposition of the hybrid microtubules in the cells (Fig. 5). Thus, cells treated with CNTs, display changes in the microtubule pattern where the radial microtubule organization is substituted by a parallel microtubule array that runs from side to side of the cytoplasm, similar to that in yeast or plant cells (Fig. 5). This change in the arrangement of the microtubule network causes an alteration in the patterns of intracellular forces. Given the importance of the centrosome as the director of the cell migratory phenomena, its disassembly generates important deleterious biomechanical effects upon force generation, that result in a loss of directionality during migration, a slower and more disordered migration all producing a significant reduction in the cellular migration speed (Fig. 6) [34–36].

**Fig. 7:**
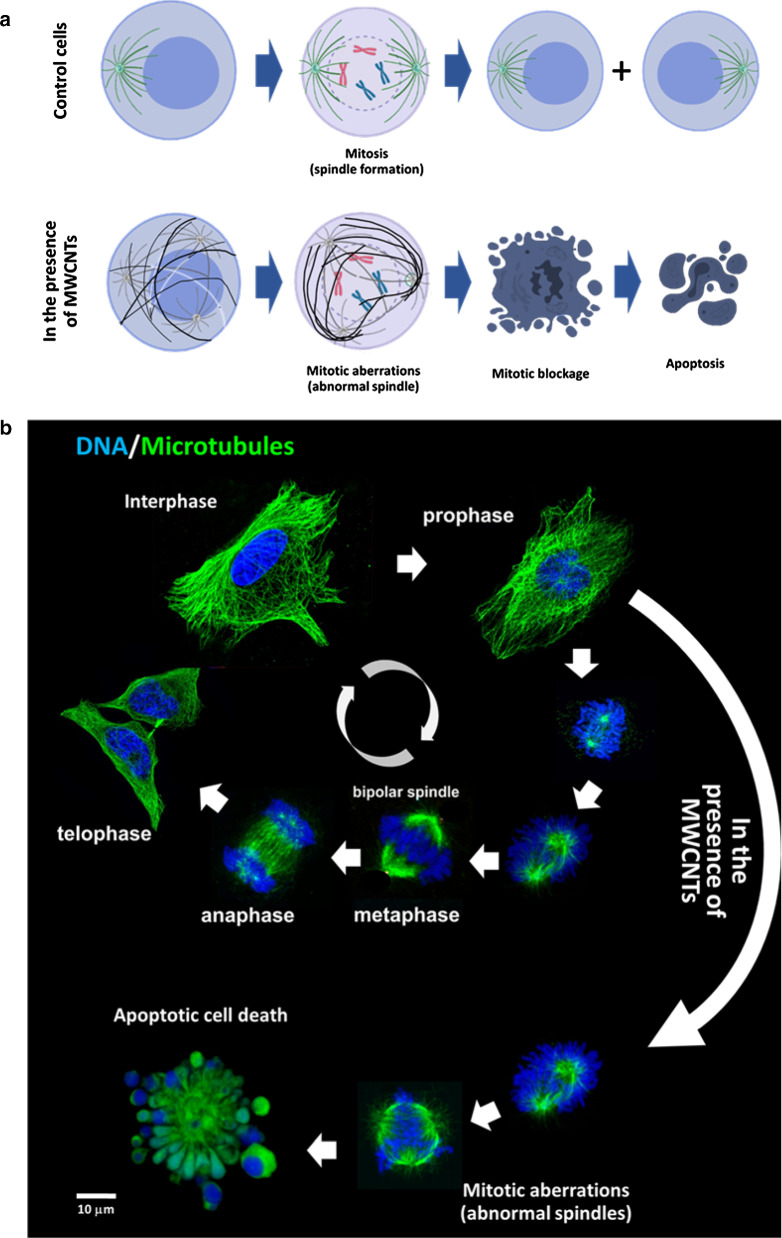
MWCNTs interference with the cell cycle.** a **Diagram comparing mitosis in untreated versus MWCNT-treated cells. Spindle formation is abnormal (apolar, tripolar, or multipolar) in the presence of MWCNTs leading to mitotic blockage and apoptosis. **b **Phases of a normal cell cycle (top) compared to that in cells treated with MWCNTs (bottom)

In proliferating cells, MWCNTs typically interfere with spindle formation. Aberrant spindles (i.e. acentrosomal or multipolar) are common in metaphase, triggering proapoptotic effects that result in the SAC activation apoptotic downstream effects. In resistant cells (i.e. cancer cells that have inhibited the apoptotic cascade) aberrant mitosis displaying aneuploidy and clastogenic effects, are common upon CNT treatment (Fig. 7).

Finally, some curiosities regarding the effect of CNTs in cellular biomechanics. MWCNTs also interfere with actin during cell division effects, typically resulting in multinucleated cells [34]. In macrophages, CNTs trigger cellular binucleation indicating that nanotubes interfere with the actomyosin contractile ring during cytokinesis, at the end of mitosis. Besides, macrophages treated with CNTs display a reduced phagocytic activity, thus supporting the idea that MWCNTs interfere with the actin cytoskeleton [34, 39]. It is also very interesting that neurons in the same cultures did not display observable deleterious changes upon exposure to CNTs, thus suggesting that cells that do not undergo intensive cytoskeletal rearrangements (such as the case of differentiated neurons) are not that much affected by nanotubes. Finally, all these remarkable effects disappear when identical doses of MWCNTs are not administered dispersed, i.e. attached to particles (500 nm diameter). No biomechanical impedance, microtubule cytoskeletal reorganization, cytotoxicity, or apoptosis is observed [60, 61], results that reinforce the hypothesis that to interact with tubulin, CNTs must be dispersed.

## MWCNTs boost the effect of microtubule-interfering drugs for cancer treatment and prevent resistance

**Fig. 8:**
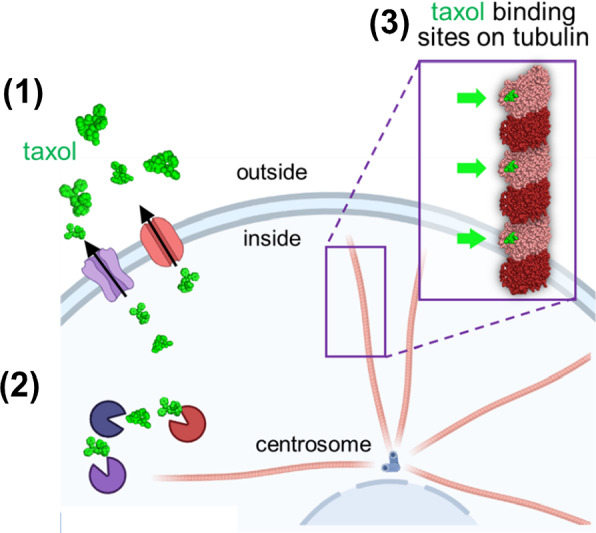
Taxol^®^(paclitaxel) interaction with microtubules and major reported resistance mechanisms. **(1)** Drug efflux pumps, **(2)** intracellular drug degradation, **(3)** tubulin mutations, or posttranslational modifications impeding microtubule-associated drug binding (green arrow)

Cell proliferation is inherent in cancer and microtubules are key elements in this process thus, tubulin is a traditional target of many antitumor therapies. Microtubule-stabilizing interfering agents-namely taxanes (paclitaxel, docetaxel, and cabazitaxel)-have become some of the most widely used and effective anticancer agents during the last 50 years with efficacy towards a broad range of cancers. These drugs behave as microtubule poisons, interfering with microtubule dynamics, inhibiting the disassembly of the tubulin polymer. To do this, these molecules bind to a lateral structural pocket localized in the polymerized β-tubulin molecule (Fig. 8). Drug interaction with the heterodimer produces structural changes in the conformation of the αβ-tubulin molecule that stabilize the protofilament, inhibiting microtubule depolymerization, also enhancing tubulin ectopic (non-centrosomal) nucleation. As a result of the treatment, dividing cells assemble aberrant spindles, often multipolar, undergoing cell cycle arrest typically at the G2/M transition phase, and finally, die by apoptosis [52, 62, 63]. However, these drugs have several critical limitations. Among these, (i) their production -they derive from natural sources and are difficult to synthesize-, (ii) their hydrophobic nature, (iii) their limited therapeutic window, (iv) their unwanted side effects, and (iv), the most worrying fact is that their chemical properties make them substrates of resistance [63, 64]. Since these drugs and carbon nanotubes similarly interfere with microtubule dynamics, it is feasible that their combined application could produce a synergistic effect to prevent resistance phenomena in cancer cells.

In general terms, there are two major resistance mechanisms to microtubule-binding drugs, those ascribed to drug efflux pumps [65], and those resulting from tubulin modifications impeding microtubule-associated drug binding [64]. In addition to these, the degradation phenomena of drugs can operate at the intracellular level (Fig. 8). Among the former, chemotherapy can be ejected from the cells through the expression of one or more energy-dependent transporters, leading to the reduction of intracellular drug levels and consequent drug insensitivity to multiple antitumoral drugs [65]. And the later resistance mechanisms include, (i) mutations in the binding site of the drug to the tubulin molecule, (ii) post-translational modifications to tubulin that interfere with drug binding, (iii) changes to the tubulin/microtubule-regulatory proteins, (iv) or even changes in the tubulin composition of microtubules. Besides, cancer cells can modify signalling pathways to exit mitosis bypassing the `security mechanisms´ (a phenomenon called mitotic slippage) thus avoiding the drug-triggered apoptotic effect [62, 66].

In this sense, the use of CNTs as drug adjuvant and/or carriers has the potential to completely transform the traditional antineoplastic treatments. CNTs, cannot be secreted out of the cells (like drugs that are small molecules) and are not easily degraded, and their microtubule-binding properties can play a pivotal effect in boosting the cytotoxic properties of the microtubule-binding drugs. CNTs associate longitudinally and intermingle with the tubulin polymers. Since they do not specifically bind to a particular tubulin type, a structural pocket, or a target site in the tubulin molecule, CNTs are not affected by tubulin mutations or posttranslational modifications that significantly interfere with tubulin-binding drugs. Consequently, CNTs are not subject to `standard´ resistance mechanisms. Indeed, some studies demonstrate that CNTs can significantly boost the antitumoral effect of taxol®, boosting its cytotoxic effects, preventing and overcoming resistance to this drug [32, 67, 68]. More interestingly, MWCNTs have also been shown to boost the effect of other antitumoral drugs such as 5-fluorouracil [11] or doxorubicin [69, 70] that are not catalogued as microtubule-poisons. These drugs can be administered in parallel to CNTs, or can be loaded using these as carriers, forming stable covalent bonds or supramolecular assemblies based on noncovalent interactions [6]. In most cases, CNTs have the carried drugs physisorbed to their nanotube surfaces by π- π stacking [11, 71].

### Finding ways for CNT administration in vivo

Finally, we want to briefly comment on how CNTs could be administered in vivo. Numerous research groups have shown how these nanomaterials-upon resuspension-, can be injected directly intravenously. Injected CNTs distribute throughout most of the organs-including the brain-, and are mainly retained in the lungs, liver, and spleen, being eliminated through the kidney and bile duct [72–75]. If CNTs are inhaled, aspirated or instilled in the pharynx or intratrachealy, the nanotubes have been reported to trigger inflammation and genotoxic effects in the lungs [76, 77]. But interestingly, and as expected by the in vitro results, the pulmonary toxicity of well-dispersed CNTs is more severe if the nanotubes are well-dispersed [73]. Alternatively, CNTs can be injected locally, i.e. intratumorally. This is how many of the studies that have served to investigate the antitumor effect of these nanomaterials have been carried out [32, 78].

## Conclusions

Carbon nanotubes, and more particularly MWCNTs, can trigger important biomechanical effects mostly resulting from their similarities to microtubules that prompt their interaction in the cells. They intermingle with the protofilaments of microtubules in living cells, stabilizing the microtubule protofilament latices, disorganizing the radial microtubule cytoskeleton. As result, MWCNTs interfere with cell migration and division, finally leading to apoptosis in highly proliferative cells. Thus, these nanomaterials behave like taxanes, some of the traditional and most successful chemotherapeutics, as a new class of microtubule-stabilizing agents.

Since, drug resistance is inherent to the nature of cancer where cells create continuously adaptation strategies-, we believe CNTs could represent a new complementary therapeutic approach against cancer cell resistance used in combination with traditional microtubule-binding drugs, such as Taxol^®^ (paclitaxel) or Epothilones, or even chemicals that operate at a different antiproliferative step, i.e. inhibiting DNA replication—such as Doxorubicin or 5-fluorouracil- to boot their effect. Moreover, MWCNTs could be used as active excipients in drug delivery systems, considering their intrinsic antitumoral properties, enhancing the therapeutic effect of traditional chemotherapy while preventing drug resistance in cancer.
